# The interventional effects of PNF training on functional ankle instability in habitually active adults

**DOI:** 10.3389/fpubh.2026.1923487

**Published:** 2026-08-26

**Authors:** Weiren Liao, Simao Xu, Rui Wang, Jingzhi Zhang

**Affiliations:** 1 College of Tourism and Sports Health, Guilin University, Guilin, China; 2 College of Physical Education and Health, Guangxi Normal University, Guilin, China; 3 Guilin Institute of Information Technology, Guilin, China

**Keywords:** balance training, functional ankle instability, habitually active adults, muscle strength, PNF training, rehabilitation

## Abstract

Functional ankle instability (FAI) is common among habitually active adults and may lead to recurrent sprains, reduced athletic performance, and long-term joint dysfunction. This randomized controlled trial aimed to compare the clinical efficacy of proprioceptive neuromuscular facilitation (PNF) training with strength-plus-balance training in habitually active adults with FAI. A total of 68 habitually active adults with FAI were randomly assigned to either a PNF training group or a strength-plus-balance training group, both undergoing an 8-week rehabilitation program. Outcome measures were assessed before and after the intervention and included the Cumberland Ankle Instability Tool (CAIT), lower limb stability tests, Star Excursion Balance Test (SEBT), and ankle muscle strength assessments. After 8 weeks, a significant group × time interaction was observed for CAIT scores (*p* < 0.001), with improvements from 13.00 ± 2.48 to 24.07 ± 3.53 in the PNF training group and from 13.47 ± 2.45 to 20.13 ± 2.72 in the strength-plus-balance training group. PNF training also produced greater gains in overall balance ability, while strength-plus-balance training was more effective for enhancing ankle inversion strength (*p* = 0.039). In conclusion, both rehabilitation programs improved ankle function in habitually active adults with FAI; however, PNF training demonstrated superior improvements in overall balance, whereas strength-plus-balance training was more effective in enhancing ankle inversion strength. These findings may assist clinicians in selecting rehabilitation strategies according to specific therapeutic goals.

**Clinical Trial Registration:**

ClinicalTrials.gov, registration number: NCT06308809.

## 1 Introduction

Ankle sprains are among the most common musculoskeletal injuries (1, 2) and are particularly prevalent in physically active populations (3, 4), accounting for approximately 16%−40% of all sports-related injuries (5–7). Approximately 40% of individuals who sustain an ankle sprain subsequently develop chronic ankle instability (CAI), characterized by persistent pain, recurrent sprains, impaired proprioception, and neuromuscular deficits (8, 9). Functional ankle instability (FAI), the most common clinical manifestation of CAI, is characterized by recurrent episodes of instability despite the absence of persistent mechanical laxity and may remain symptomatic for years after the initial injury (10).

Research indicates that approximately 40% of traumatic ankle injuries and nearly half of ankle sprains occur during sports activities (4, 11, 12), with the highest incidence rates observed in basketball (41.1%), American football (9.3%), and soccer (7.9%) (13–15). There is increasing evidence supporting the use of exercise therapy as a primary component of treatment plans (16–19), and its effectiveness has been confirmed, especially when initiated early after acute ankle sprains (20). Individuals with FAI exhibit insufficient ankle muscle strength and reduced balance ability (21, 22). As a result, scholars have designed corresponding ankle muscle strength training and balance training programs based on the characteristics of FAI (23–28). Nam et al. found that 8 weeks of balance training incorporating knee joint movements significantly produced significantly greater increases than conventional strength training alone in the electromyographic activity of the tibialis anterior, peroneus longus, and lateral gastrocnemius muscles in adults with FAI, suggesting enhanced neuromuscular control and ankle stability (29). Similarly, Park et al. implemented a six-week strength-plus-balance training program in adolescent soccer players with FAI and reported significantly greater improvements compared with strength training in Cumberland Ankle Instability Tool (CAIT) scores, ankle dorsiflexion and inversion strength, static balance, and performance measures (30). More recently, Xu et al. used ankle balance device training programs in individuals with FAI and observed significant improvements compared with conventional strength-based rehabilitation in peroneal muscle reaction time, ankle inversion and eversion torque, and postural control ability (31).

FAI has been classically distinguished from mechanical laxity since the seminal work of Freeman and colleagues in the early 1960s. Freeman demonstrated that ankle instability may persist even in the absence of objective ligament laxity and proposed that impaired ankle joint mechanoreceptor function and disrupted reflexive neuromuscular control are key contributors to functional instability (32–36). This conceptual framework highlighted that FAI is not solely a consequence of structural impairment but also involves deficits in proprioception and sensorimotor control, providing an important theoretical basis for rehabilitation strategies targeting functional recovery.

However, although exercise therapy has been widely recognized as an effective rehabilitation strategy for FAI, recent systematic reviews and meta-analyses have indicated that most rehabilitation approaches have primarily focused on isolated components of ankle function, such as strength, balance, or proprioceptive training, rather than comprehensive neuromuscular control (37). However, FAI involves complex impairments in neuromuscular coordination, postural control, and dynamic stability, suggesting that rehabilitation strategies incorporating broader movement control demands may be beneficial. Contemporary reviews have emphasized that rotational ankle instability and altered sensorimotor integration play critical roles in recurrent sprains and persistent functional limitations. Therefore, interventions that address multiple dimensions of neuromuscular function may represent an important direction for optimizing rehabilitation outcomes in individuals with FAI (38). Recent advances in functional assessment have further emphasized that ankle instability is a complex and multidimensional disorder involving interactions among biomechanical characteristics, movement patterns, and sensorimotor control rather than isolated impairments. Emerging approaches integrating multiple functional features and data-driven analysis may provide more comprehensive perspectives for identifying and evaluating ankle dysfunction (39). However, current rehabilitation studies in individuals with FAI have primarily focused on individual components such as muscle strength, balance, or proprioceptive function, and comprehensive interventions targeting integrated neuromuscular control remain insufficiently explored.

Proprioceptive neuromuscular facilitation (PNF) is a rehabilitation approach that aims to enhance neuromuscular control, coordination, and joint stability through spiral–diagonal movement patterns combined with proprioceptive stimulation and resistance. By facilitating coordinated activation of agonist and antagonist muscle groups, PNF may improve motor unit recruitment, joint mobility, and dynamic postural control. Given that FAI is characterized by impaired sensorimotor control and reduced dynamic stability, PNF-based interventions may represent a promising rehabilitation strategy for restoring functional ankle stability. Previous research has demonstrated the benefits of neuromuscular and proprioceptive training methods derived from PNF principles in individuals with CAI, particularly those with functional deficits related to FAI. For example, Yesilkir and Ergezen Sahin compared PNF, balance training, and conventional physiotherapy in amateur athletes with chronic ankle instability, finding that PNF significantly enhanced postural control, functional performance, and neuromuscular coordination compared to standard physiotherapy (40). Similarly, Harmouche et al. reported substantial improvements in functional outcomes, balance, and perceived ankle stability following a PNF protocol, underscoring the critical role of proprioceptive activation in preventing recurrent ankle injuries (41). Although both PNF and strength-plus-balance training have demonstrated beneficial effects in individuals with FAI, direct comparisons between these interventions using randomized controlled designs remain scarce. Therefore, further research is needed to determine whether PNF provides additional benefits over conventional strength-plus-balance training for improving functional stability and rehabilitation outcomes in individuals with FAI.

This study aimed to compare the clinical efficacy of PNF training and strength-plus-balance training for treating FAI in habitually active adults. This population was selected because habitually active adults frequently participate in recreational or non-professional physical activities, placing them at increased risk of ankle injury while differing from competitive athletes, who often receive structured sports medicine support, and sedentary individuals, who experience lower levels of repetitive mechanical loading. This group represents an important target population for early rehabilitation, as persistent instability may restrict physical activity participation, increase recurrent injury risk, and contribute to long-term functional impairment. Given prior evidence that PNF training enhances joint proprioception, coordination, and dynamic balance, whereas strength-plus-balance training improves muscle strength and postural control, we hypothesized that PNF training would produce greater improvements in ankle stability, balance, and functional performance compared with strength-plus-balance training in individuals with FAI.

## 2 Materials and methods

### 2.1 Research design

This randomized controlled trial was designed and reported in accordance with the CONSORT statement. This prospective randomized controlled trial was conducted from September 2024 to December 2024. The study received ethical approval from the Ethics Committee of Guangxi Normal University (approval number: 20230806009). The study protocol was prospectively registered with ClinicalTrials.gov PRS on March 14, 2024 (registration number: NCT06308809), prior to the commencement of participant recruitment.

### 2.2 Participants

The sample size for this study was calculated using G^*^Power 3.1.9.7 software. Based on a review of similar previous studies that used the CAIT score as the primary outcome measure, the effect size (f) was determined to be 0.25 (42, 43). With α set at 0.05 and a power of 0.95, the required sample size was calculated as 54 participants. Assuming an equal allocation ratio of 1:1 between the PNF training group and the strength-plus-balance training group, and considering a 20% dropout rate (30), the final sample size was increased to 68 participants. Volunteers were recruited from Guangxi Normal University and Guilin University through advertisements and social media. All participants provided written informed consent, affirming their ability to meet the experimental requirements.

Eligible volunteers with relevant symptoms were enrolled via an online system used for inclusion, randomization, and data collection, limited to preliminary investigation by the researchers. Post- recruitment, diagnosis of FAI completed by the blinded researchers for the chief orthopedic physician of Guilin Municipal Hospital of Traditional Chinese Medicine, using the International Ankle Consortium guidelines for CAI screening (44).

Participants were required to meet the following inclusion criteria (45, 46):

(1) Regular participation in physical activities, including basketball, soccer, running, fitness training, aerobic exercise, or school-based physical conditioning programs, for at least 1–2 h per day and 2–3 times per week.
(2) Diagnosis of unilateral functional ankle instability (FAI) by an orthopedic physician according to the International Ankle Consortium guidelines (44), including: a history of recurrent ankle instability episodes; a Cumberland Ankle Instability Tool (CAIT) score <24; absence of mechanical ankle instability.
(3) History of at least one mild-to-moderate ankle sprain (Grade I–II) within the previous 12 months.
(4) History of at least two episodes of ankle joint “giving way” or loss of control within the previous 6 months.
(5) No formal or informal rehabilitation treatment for ankle instability within the previous month.
(6) No evidence of mechanical ankle instability, as determined by negative anterior drawer and talar tilt tests.
(7) Exercise-related ankle pain ≤ 5 points on the Visual Analog Scale (VAS) during or after physical activity.
(8) Age between 18 and 23 years.

Exclusion criteria were as follows:

(1) Presence of concurrent injuries, previous surgery, or fractures involving the trunk or lower extremities.
(2) Ankle pain caused by infectious diseases or other non-mechanical conditions.
(3) Severe neurological disorders or soft tissue injuries surrounding the ankle joint.
(4) Instability involving joints other than the ankle.

Participants were withdrawn from the study if they met any of the following dropout criteria:

(1) Accidental injuries occurring during the intervention period.
(2) Poor adherence to the training protocol.
(3) Incomplete outcome data.
(4) Any condition that prevented continued participation in the intervention.

During the intervention period, participants were instructed not to engage in any additional ankle-specific rehabilitation programs or high-intensity training beyond the study protocol. The use of analgesic or anti-inflammatory medications was not permitted during the intervention period. However, habitual daily physical activities and required academic physical education classes were allowed, provided that they did not involve additional ankle rehabilitation or high-intensity loading.

### 2.3 Intervention

**Figure 1:**
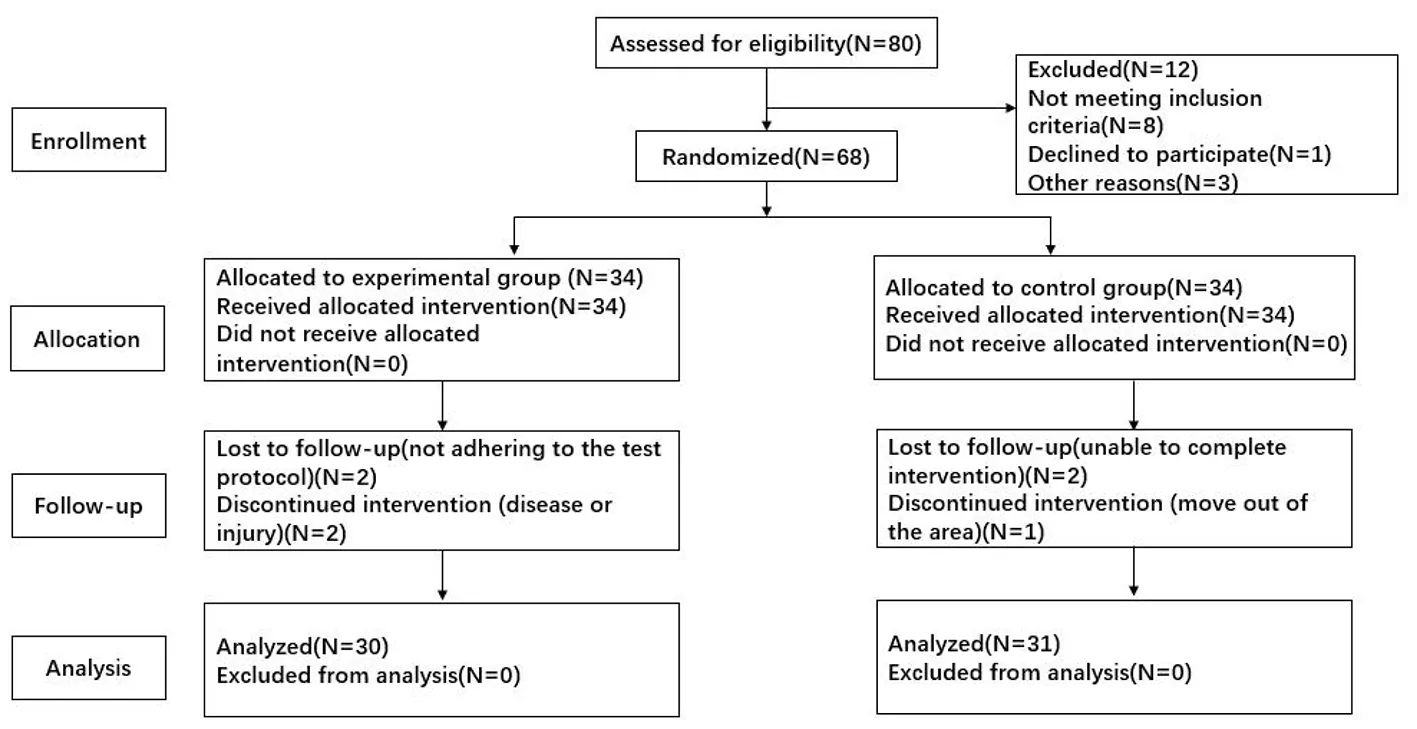
CONSORT flow chart.

Due to the CONSORT guidelines enabling a more thorough and transparent reporting of randomized controlled trials (46), the sampling process is illustrated in Figure 1, which also details the sampling procedure.

Sixty-eight participants were randomly allocated to the PNF training group (*n* = 34) or the strength-plus-balance training group (*n* = 34). The random allocation sequence was generated using random number table, and allocation concealment was achieved using sequentially numbered, opaque, sealed envelopes. The experimental group received PNF training, whereas the control group received strength-plus-balance training. Due to the nature of exercise interventions, participants and intervention administrators could not be blinded to group allocation. However, outcome assessors and data collectors were blinded to participant group assignments to minimize potential measurement bias.

The exercise intervention protocol was designed by the researchers for this study and was implemented by rehabilitation therapists who were unaware of the specific group allocations of the experimental and control groups. The exercise intervention sessions were fixed between 6 PM and 10 PM, conducted in a rehabilitation room with consistent temperature and humidity conditions. Both the training and data collection were overseen by the same rehabilitation therapist and data collector, respectively, to control for bias introduced by covariates.

### 2.4 Training program

The training program was developed according to established rehabilitation protocols and widely accepted clinical practices for FAI (47). To ensure its scientific authenticity and validity, the program was designed and reviewed by a team of licensed rehabilitation therapists with extensive clinical experience in musculoskeletal rehabilitation. The exercise content and progression were adapted from evidence-based practices commonly applied in sports rehabilitation, as supported by Lazarou et al. (47), ensuring both safety and clinical relevance for the participants.

Participants in both the experimental and control groups underwent an 8-week rehabilitation training program, conducted three times per week (Monday, Wednesday, and Friday) at the Sports Rehabilitation Room, Guangxi Normal University. Each rehabilitation session lasted approximately 40–45 min. Before each session, participants performed a 5-min warm-up targeting the ankle and lower leg muscles, followed by 30–35 min of the respective rehabilitation training, and concluded with a 5-min cool-down targeting the ankle and lower leg muscles.

The training programs for both groups followed a structured progressive overload protocol throughout the 8-week intervention period. Exercise intensity and training volume were progressively increased according to predefined criteria, including increases in resistance, exercise duration, number of sets, and movement complexity, while ensuring proper movement quality. In the PNF training group, participants performed two PNF-based ankle training approaches (isometric combination training and stability reversal training) designed to enhance neuromuscular coordination, joint stability, and muscle activation patterns. In the strength-plus-balance training group, participants performed elastic resistance band–based ankle strengthening exercises (dorsiflexion, plantarflexion, inversion, and eversion) combined with balance-oriented single-leg standing and catching exercises targeting ankle muscle strength and postural control. Elastic bands with progressive resistance levels were used, and resistance was increased when participants completed the prescribed repetitions with correct technique and without compensatory movements. Detailed training parameters, including exercise type, intensity, contraction duration, sets, rest intervals, and weekly progression criteria, are presented in Table 1.

**Table 1:** Training schedule for experimental and control groups.

Group	Exercise name	Week 1–2				Week 3–4				Week 5–6				Week 7–8			
		Duration (S)	Sets (sets)	Rest Between Sets (S)	Frequency (times/week)	Duration (S)	Sets (sets)	Rest between sets (S)	Frequency (times/week)	Duration (S)	Sets (sets)	Rest between sets (S)	Frequency (times/week)	Duration (S)	Sets (sets)	Rest between sets (S)	Frequency (times/week)
Experimental group	Isometric combination training	5	3	90	3	5	4	90	3	8	4	60	3	8	4	60	3
	Stability reversal training	5	3	90	3	5	4	90	3	8	4	60	3	8	4	60	3
Control group	Strength training (dorsiflexion)	10	4	50	3	10	5	38	3	14	5	34	3	15	6	25	3
	Strength training (plantarflexion)	10	4	50	3	10	5	38	3	14	5	34	3	15	6	25	3
	Strength training (inversion)	10	4	50	3	10	5	38	3	14	5	34	3	15	6	25	3
	Strength training (eversion)	10	4	50	3	10	5	38	3	14	5	34	3	15	6	25	3
	Single leg stand catch and throw training	90	3	90	3	120	3	60	3	90	4	60	3	100	4	35	3

Across both groups, the training programs were designed following standardized principles of frequency, intensity, and volume, with a fixed frequency of three sessions per week over 8 weeks and a structured, progressive increase in training load.

### 2.5 Experimental group training content

#### 2.5.1 PNF isotonic combined training

Participants removed their shoes and socks and lay supine on the rehabilitation bed. The therapist stood beside the affected side of the participant in a lunge stance. Starting from the initial position of the lower limb extension pattern, with the ankle inverted and the hip externally rotated, the therapist placed one hand in a pincer grip on the dorsum of the participant's foot and used the heel of the other hand to support the participant's heel while applying resistance. The therapist guided the participant to perform concentric contractions against the resistance until reaching the terminal position without pausing. The therapist then adjusted the resistance in the opposite direction, guiding the participant to perform eccentric contractions against the resistance until returning to the starting position.

#### 2.5.2 PNF stability inversion training

Participants removed their shoes and socks and lay supine on the rehabilitation bed. The therapist stood beside the affected side of the participant in a lunge stance. Starting from the initial position of the lower limb flexion pattern, the therapist applied resistance to the dorsum of the participant's foot with one hand and to the participant's knee with the other hand. The therapist guided the participant to perform concentric contractions against the resistance until reaching the terminal position. The participant then maintained the position against the resistance, achieving isometric muscle contraction for stability. After reaching the stable position, the therapist guided the participant to return to the initial position through eccentric contractions.

### 2.6 Control group training content

#### 2.6.1 Resistance band training

Participants removed their shoes and socks and lay supine on the rehabilitation bed, with their hands naturally placed on their chest and the affected leg kept straight. For ankle dorsiflexion against resistance, the therapist held both ends of the resistance band and looped it around the dorsum of the participant's foot, guiding the participant to move against the resistance to the terminal position. For ankle plantarflexion against resistance, the participant held both ends of the resistance band and looped it around the sole of the foot, moving against the resistance to the terminal position. For ankle inversion against resistance, the therapist held both ends of the resistance band and looped it around the inner side of the participant's foot, guiding the participant to move against the resistance to the terminal position. For ankle eversion against resistance, the therapist held both ends of the resistance band and looped it around the outer side of the participant's foot, guiding the participant to move against the resistance to the terminal position.

#### 2.6.2 Single leg stand and catch training

Participants stood on one leg on the affected side, maintaining balance, with their hands ready to catch a ball. The therapist stood six meters away, throwing the ball toward the participant's front, left side (0.5–1 meters), and right side (0.5–1 meters). The participant caught the ball and threw it back to the therapist, quickly regaining balance after each catch. If the ball was dropped, the participant continued with the single-leg stand and catch training without retrieving the ball.

### 2.7 Testing program

The primary outcome was the CAIT score, which was used to evaluate functional ankle instability. Secondary outcomes included lower limb stability, dynamic balance assessed by the Star Excursion Balance Test (SEBT), and ankle muscle strength. Baseline testing was conducted within 2 days prior to the start of the intervention. All tests were conducted in the order of CAIT → lower limb stability → SEBT → muscle strength for all participants to ensure consistency. All outcome measures were assessed twice — once before the start of the 8-week intervention (pre-test) and once within 2 days after completion of the final training session (post-test).

#### 2.7.1 CAIT score

The CAIT consists of nine items with a total score of 30 points. In this study, a Chinese-translated version of the CAIT was used because all participants were Chinese-speaking and completed the questionnaire in their native language. Before administration, detailed instructions were provided to ensure that participants understood each item. A score of ≤ 24 indicates FAI. The CAIT scores were calculated, recorded, and saved. If any mistakes were made in completing the questionnaire, it was filled out again, and the corrected scores were recorded. The CAIT has demonstrated high test-retest reliability (ICC > 0.9) in assessing functional ankle instability (48).

#### 2.7.2 Lower limb stability test

The Micro Swing 6.0 center of gravity movement track tester was used for this test. Participants were first familiarized with the testing system and environment and trained in the testing methods. They then stood barefoot on one leg at the center of the balance measurement device, with their hands naturally placed by their sides, maintaining balance for 30 s. Data were recorded by the testing system. The measurement was repeated three times, with a 5-min interval between each test. Data collected included swing frequency and stability coefficient, with the best result from the three tests recorded. The Micro Swing 6.0 balance tester has shown excellent reliability in previous studies assessing postural stability (49).

#### 2.7.3 Star excursion balance test

**Figure 2:**
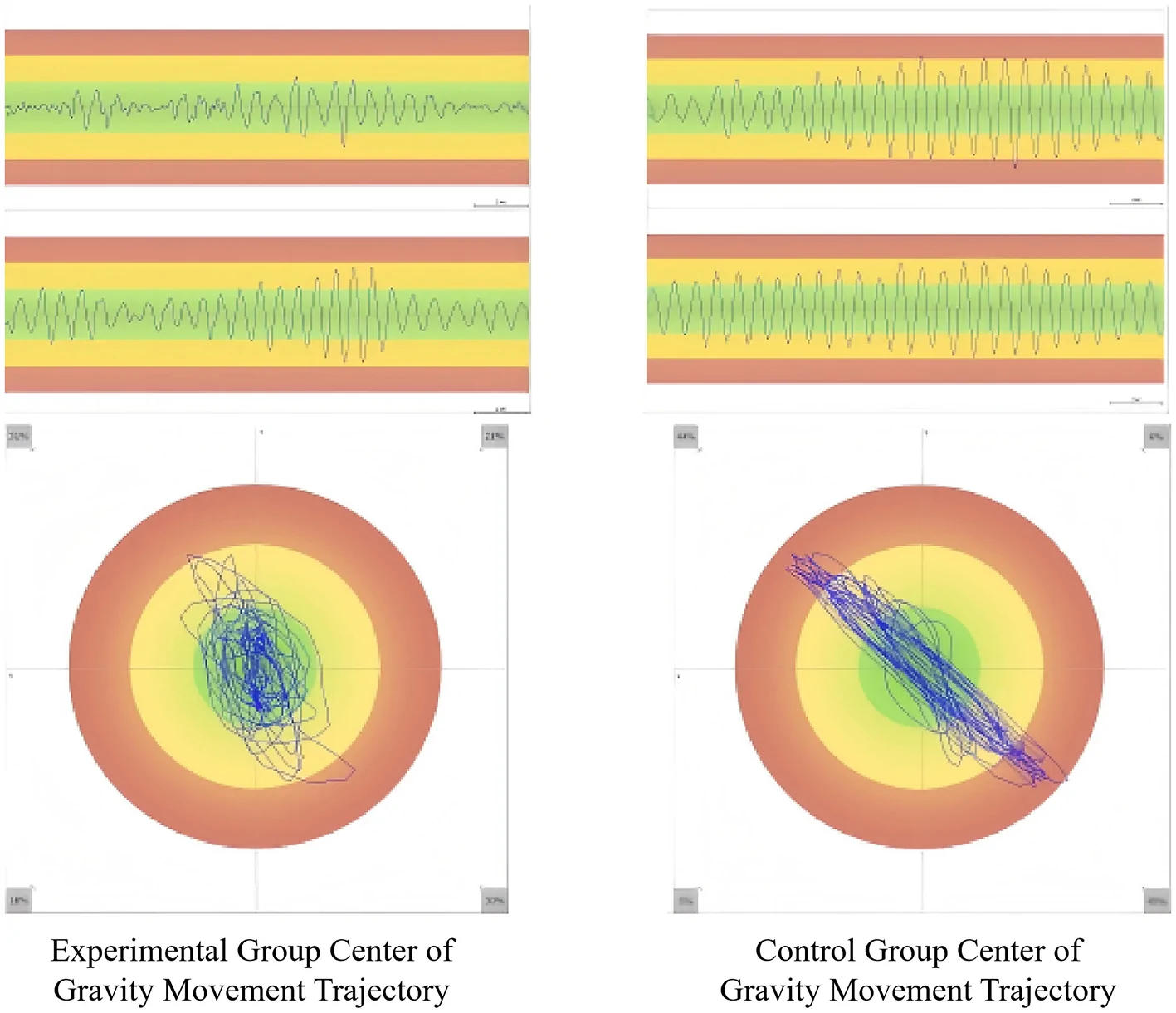
Diagram of lower limb stability changes in both groups before and after training.

The SEBT was used to assess balance (50). Participants were instructed on the SEBT method and allowed a practice trial. The length of the participants‘ lower limbs (from the anterior superior iliac spine to the medial malleolus) was measured and recorded. Participants stood barefoot on the central point of the star pattern with the affected foot as the supporting leg, reaching as far as possible in eight directions: anterior, anterolateral, lateral, posterolateral, posterior, posteromedial, medial, and anteromedial (as shown in Figure 2). For the left supporting leg, the directions were reached in a counterclockwise sequence; for the right supporting leg, the directions were reached in a clockwise sequence. After each reach, participants returned the non-supporting foot to the starting position and rested for 5 s. Reach distances were recorded, and the measurement was repeated three times to obtain the maximum reach distance. The maximum reach distances in each direction were standardized relative to the participants' leg length and recorded. The SEBT demonstrates high reliability and validity for dynamic balance assessment.

#### 2.7.4 Muscle strength test

The MicroFET2 handheld muscle strength tester was used for muscle strength testing, expressed in pounds (LB). Participants were familiarized with the testing system and environment and trained in the testing methods. They then removed their shoes and socks and lay supine on the rehabilitation bed, with their hands naturally placed on their chest. The muscle strength of ankle dorsiflexion, plantarflexion, inversion, and eversion was tested and recorded. Each movement direction was measured five times. Data collected included the muscle strength of ankle dorsiflexion, plantarflexion, inversion, and eversion. Each movement was assessed five times, and the highest value among the five trials was recorded to represent the participants' maximal voluntary isometric strength. This approach was selected because handheld dynamometer assessments of muscle strength are commonly used to evaluate peak force production, and the highest value may better reflect maximal neuromuscular capacity when participants are adequately familiarized with the testing procedure. The handheld dynamometer has been reported to have good intra-rater and inter-rater reliability in assessing ankle strength (49).

### 2.8 Data analysis

Test data are expressed as mean ± standard deviation (SD). Statistical analyses of the test data were performed using PASW Statistics 24.0 software. The Shapiro–Wilk test was used to verify data normality, and all variables met the assumption of normal distribution. To examine the effects of intervention over time and between groups, a mixed repeated-measures analysis of variance (RM-ANOVA) was conducted for each outcome variable, with time (pre-intervention vs. post-intervention) as the within-subjects factor and group (PNF training vs. strength-plus-balance training) as the between-subjects factor. The primary effects of interest included the main effects of time and group, as well as the group × time interaction. When a significant interaction effect was identified, *post hoc* pairwise comparisons with appropriate adjustments were performed to further explore within- and between-group differences. A *p*-value of less than 0.05 was considered statistically significant for all analyses.

## 3 Results

### 3.1 Participant characteristics

Of the 68 randomized participants, seven participants discontinued the study during the intervention period, resulting in a dropout rate of 10.3%. The reasons for dropout are presented in Figure 1. A per-protocol analysis was performed, and data from the 61 participants who completed the intervention and post-intervention assessments were included in the final analysis. No significant differences were observed in baseline characteristics between participants who completed the study and those who withdrew. The general characteristics of the subjects in the experimental and control groups are shown in Table 2. No significant baseline differences were observed in age, height, weight, body mass, body mass index (BMI), or duration of FAI between the two groups (*p* > 0.05).

**Table 2:** General information of subjects.

Test group	Age (years)	Height (m)	Body mass (kg)	BMI (kg/m^2^)
EG (*n* = 30)	20.58 ± 1.85	1.77 ± 0.05	72.68 ± 6.52	23.19 ± 3.64
CG (*n* = 31)	21.35 ± 2.32	1.76 ± 0.05	69.75 ± 7.16	22.52 ± 3.41
*t*	−1.314	1.512	1.435	1.254
*p*	0.768	0.313	0.275	0.801

EG, experimental group; CG, control group.

### 3.2 Outcome measures

#### 3.2.1 Effect of PNF and strength-plus-balance training on CAIT score

**Figure 3:**
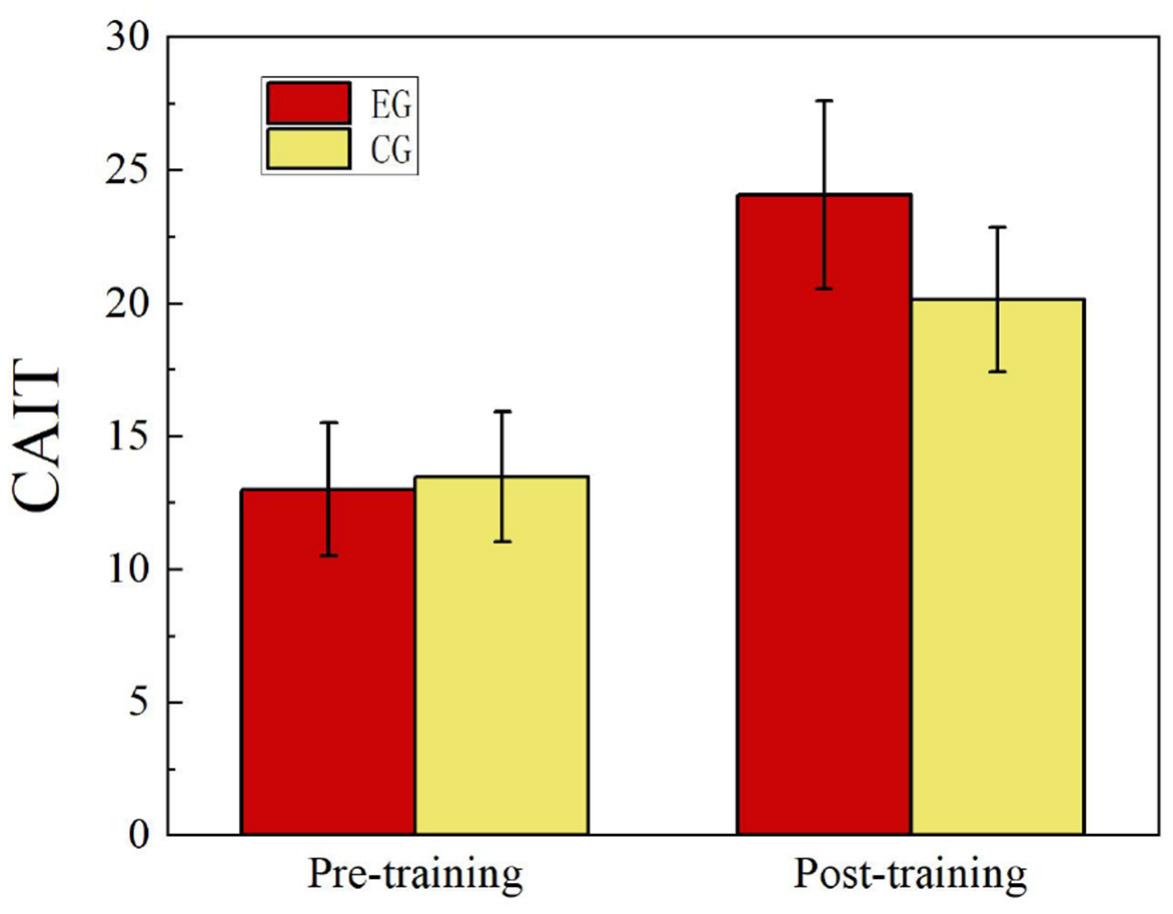
CAIT score statistics for both groups. EG, experimental group; CG, control group.

Pre- and post-training, both groups completed the CAIT self-assessment questionnaire. Baseline CAIT scores did not differ significantly between groups (*p* > 0.05). Mixed repeated-measures ANOVA revealed a significant main effect of time and a significant group × time interaction for CAIT scores (*p* < 0.001), indicating significant improvements in both groups, with greater improvement in the PNF training group (Table 3, Figure 3).

**Table 3:** CAIT score statistics for both groups.

Group	Pre-training score	Post-training score	*t*1	*p*1
EG (*n* = 30)	13.00 ± 2.48	24.07 ± 3.53	−19.902	0.000
CG (*n* = 31)	13.47 ± 2.45	20.13 ± 2.72	−15.811	0.000
*t*	−0.519	3.415	–	–
*p*	0.608	0.002	–	–

*t*1 and *p*1 for within-group changes reflect the main effect of time, while t and p-values for between-group differences reflect the group × time interaction effect. EG, experimental group; CG, control group.

#### 3.2.2 Effect of PNF and strength-plus-balance training on ankle muscle strength

**Figure 4:**
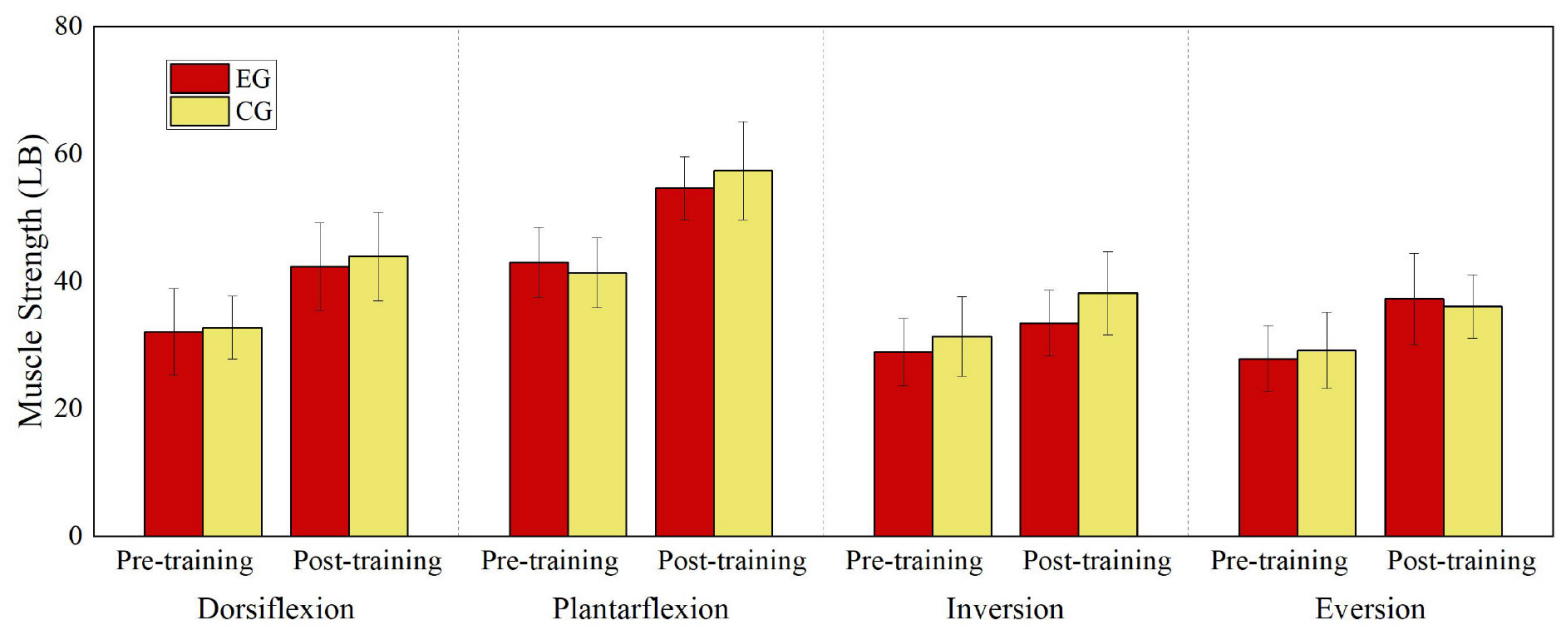
Muscle strength test results for both groups. EG, experimental group; CG, control group.

Ankle muscle strength was tested in both groups before and after the intervention. No significant baseline differences were observed between groups for dorsiflexion, plantarflexion, inversion, or eversion strength (*p* > 0.05). Mixed repeated-measures ANOVA demonstrated significant main effects of time for all ankle muscle strength outcomes (all *p* < 0.05). A significant group × time interaction was observed only for inversion strength (*p* < 0.05), with greater post-intervention gains in the strength and balance training group, whereas no significant interaction effects were found for dorsiflexion, plantarflexion, or eversion (Table 4, Figure 4).

**Table 4:** Muscle strength test results for both groups.

Direction	Group	Pre-training (LB)	Post-training (LB)	*t*1	*p*1
Dorsiflexion	EG (*n* = 30)	32.09 ± 6.79	42.28 ± 6.88	−7.863	0.000
	CG (*n* = 31)	32.77 ± 4.91	43.92 ± 6.90	−7.340	0.000
	*t*	−0.311	−0.652	–	–
	*p*	0.758	0.520	–	–
Plantarflexion	EG (*n* = 30)	42.98 ± 5.49	54.59 ± 4.94	−12.737	0.000
	CG (*n* = 31)	41.35 ± 5.50	57.35 ± 7.71	−9.645	0.000
	*t*	0.814	−1.164	–	–
	*p*	0.423	0.256	–	–
Inversion	EG (*n* = 30)	28.92 ± 5.28	33.47 ± 5.17	−14.974	0.000
	CG (*n* = 31)	31.37 ± 6.28	38.14 ± 6.53	−14.891	0.000
	*t*	−1.155	−2.171	–	–
	*P*	0.258	0.039	–	–
Eversion	EG (*n* = 30)	27.87 ± 5.22	37.21 ± 7.15	−9.054	0.000
	CG (*n* = 31)	29.17 ± 5.96	36.07 ± 4.98	−8.869	0.000
	*t*	−0.635	0.504	–	–
	*p*	0.531	0.619	–	–

*t*1 and *p*1 for within-group changes reflect the main effect of time, while *t* and *p*-values for between-group differences reflect the group × time interaction effect. EG, experimental group; CG, control group.

#### 3.2.3 Effect of PNF and strength-plus-balance training on lower limb stability

**Figure 5:**
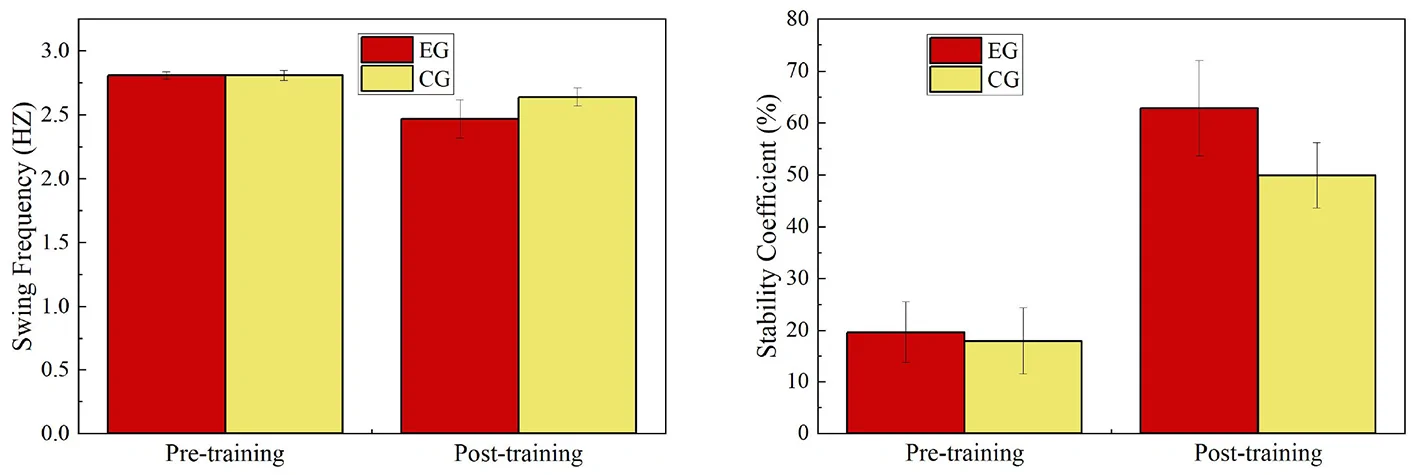
Trajectory of center of gravity movement test results for both groups. EG, experimental group; CG, control group.

Lower limb stability was tested in both groups before and after the intervention. Baseline values of Swing Frequency and Stability Coefficient were comparable between groups (*p* > 0.05). Mixed repeated-measures ANOVA revealed significant main effects of time and significant group × time interaction effects for both parameters (*p* < 0.05), indicating improvements in balance ability in both groups, with greater improvements following PNF training (Table 5, Figure 5).

**Table 5:** Trajectory of center of gravity movement test results for both groups.

Indicator	Group	Pre-training	Post-training	*t*1	*p*1
Swing frequency (HZ)	EG (*n* = 30)	2.81 ± 0.03	2.47 ± 0.15	9.056	0.000
	CG (*n* = 31)	2.81 ± 0.04	2.64 ± 0.07	9.539	0.000
	*t*	−0.592	−4.026	–	–
	*p*	0.559	0.000	–	–
Stability coefficient (%)	EG (*n* = 30)	19.60 ± 5.90	62.80 ± 9.17	−20.840	0.000
	CG (*n* = 31)	17.93 ± 6.40	49.87 ± 6.29	−44.338	0.000
	*t*	0.742	4.506	–	–
	*p*	0.465	0.000	–	–

*t*1 and *p*1 for within-group changes reflect the main effect of time, while *t* and *p*-values for between-group differences reflect the group × time interaction effect. EG, experimental group; CG, control group.

#### 3.2.4 Effect of PNF and strength-plus-balance training on SEBT

**Figure 6:**
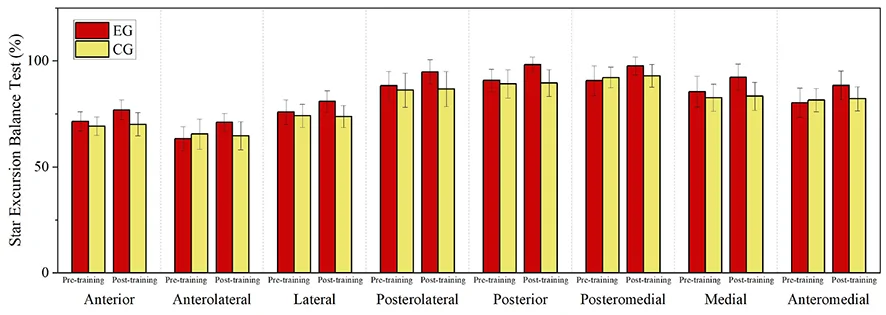
Star excursion balance test results for both groups. EG, experimental group; CG, control group.

The SEBT was conducted on both groups before and after the intervention. No significant baseline differences were observed between groups across any SEBT direction (*p* > 0.05). Mixed repeated-measures ANOVA demonstrated significant main effects of time and significant group × time interaction effects across all SEBT directions (*p* < 0.05). *Post hoc* analyses showed significant improvements in the PNF training group, whereas no significant time effects were observed in the control group, indicating that PNF training was more effective in improving dynamic balance ability (Table 6, Figure 6).

**Table 6:** Star excursion balance test results for both groups.

Direction	Group	Pre-training (%)	Post-training (%)	*t*1	*p*1
Anterior	EG (*n* = 30)	71.47 ± 4.52	76.93 ± 4.64	−9.771	0.000
	CG (*n* = 31)	69.27 ± 4.40	70.13 ± 5.40	−1.148	0.270
	*t*	1.351	3.702	–	–
	*p*	0.187	0.001	–	–
Anterolateral	EG (*n* = 30)	63.33 ± 5.61	71.07 ± 4.20	−12.614	0.000
	CG (*n* = 31)	65.53 ± 7.15	64.73 ± 6.68	0.933	0.367
	*t*	−0.937	3.108	–	–
	*p*	0.357	0.005	–	–
Lateral	EG (*n* = 30)	75.93 ± 5.78	80.93 ± 5.04	−9.682	0.000
	CG (*n* = 31)	74.13 ± 5.40	73.73 ± 5.27	0.612	0.550
	*t*	0.882	3.826	–	–
	*p*	0.385	0.001	–	–
Posterolateral	EG (*n* = 30)	88.33 ± 6.75	94.87 ± 5.72	−9.676	0.000
	CG (*n* = 31)	86.27 ± 8.06	86.73 ± 8.27	−0.777	0.450
	*t*	0.762	3.134	–	–
	*p*	0.453	0.004	–	–
Posterior	EG (*n* = 30)	90.80 ± 5.28	98.20 ± 3.55	−11.329	0.000
	CG (*n* = 31)	89.13 ± 6.67	89.60 ± 6.39	−0.714	0.487
	*t*	0.758	4.557	–	–
	*p*	0.455	0.000	–	–
Posteromedial	EG (*n* = 30)	90.67 ± 7.05	97.60 ± 4.24	−5.506	0.000
	CG (*n* = 31)	92.20 ± 4.87	92.93 ± 5.38	−1.140	0.274
	*t*	−0.693	2.639	–	–
	*p*	0.494	0.014	–	–
Medial	EG (*n* = 30)	85.53 ± 7.21	92.33 ± 6.16	−10.364	0.000
	CG (*n* = 31)	82.67 ± 6.32	83.33 ± 6.60	−1.085	0.296
	*t*	1.158	3.862	–	–
	*p*	0.257	0.001	–	–
Anteromedial	EG (*n* = 30)	80.27 ± 6.91	88.40 ± 6.81	−14.114	0.000
	CG (*n* = 31)	81.53 ± 5.57	82.20 ± 5.71	−1.113	0.284
	*t*	−0.553	2.702	–	–
	*p*	0.585	0.012	–	–

*t*1 and *p*1 for within-group changes reflect the main effect of time, while *t* and *p*-values for between-group differences reflect the group × time interaction effect. EG, experimental group; CG, control group.

## 4 Discussion

This randomized controlled trial demonstrated that both PNF-based training and strength-plus-balance training improved functional outcomes in physically active adults with FAI, although PNF produced greater improvements in perceived ankle stability and balance performance, whereas strength-plus-balance training showed relatively greater effects on specific ankle muscle strength outcomes. These findings suggest that both rehabilitation approaches may be effective for FAI management, but their benefits may differ depending on the primary therapeutic target. From a theoretical perspective, the superior effects of PNF training on functional stability and balance are consistent with the classical sensorimotor framework proposed by Freeman and the contemporary concept of rotational ankle instability. By emphasizing spiral–diagonal movement patterns and integrated neuromuscular control across the ankle, knee, and hip, PNF may target deficits extending beyond isolated ankle strength and address key mechanisms underlying functional ankle instability (32–36).

### 4.1 Considerations for selecting exercise intervention programs

Rehabilitation treatment for FAI primarily involves exercise therapy, tailored to the mechanisms underlying functional deficits. Many previous studies have reported that individuals with functional ankle instability often present with deficits in ankle muscle strength and reduced balance ability (21). Therefore, researchers have developed ankle strength-plus-balance training programs to address these impairments, with the aim of improving muscle strength, postural control, and functional stability. Such targeted rehabilitation programs have demonstrated measurable improvements in symptoms, balance performance, and muscle strength among individuals with ankle instability (51).

Research has suggested that individuals with FAI not only exhibit local ankle muscle strength deficits and reduced balance ability but may also demonstrate impairments in lower-limb sensorimotor function. However, traditional rehabilitation training for FAI has primarily focused on improving local ankle muscle strength and balance, with less emphasis on integrated lower-limb movement control. Therefore, comprehensive lower-limb training may provide additional benefits for individuals with FAI. Yang et al. investigated PNF in individuals with ankle instability and reported improvements in postural control; their study involved recreationally active young adults and used progressive PNF training over several weeks (52). PNF techniques have been widely applied in the rehabilitation of functional impairments involving joints and peripheral nerves, potentially offering advantages in improving coordinated movement patterns and functional recovery. Unlike traditional rehabilitation approaches that primarily target local joint impairments, PNF emphasizes spiral-diagonal movement patterns and integrated limb control, which may facilitate broader neuromuscular adaptations (53). In the present study, isotonic combination training and stability reversal training based on PNF lower-limb patterns were applied, and the observed improvements in ankle stability may be related to enhanced proprioceptive input and neuromuscular control. Therefore, improvements in FAI rehabilitation should not be interpreted solely as changes in individual outcomes, but rather as the restoration of an integrated functional system involving muscle capacity, postural control, and sensorimotor coordination. In this context, PNF-based training may influence multiple components of functional recovery through coordinated multi-joint movement patterns and task-specific neuromuscular challenges.

The strength-plus-balance training group received a rehabilitation program primarily focusing on local lower-limb function. Resistance band training may effectively increase ankle muscle strength and contribute to improved ankle stability. Single-leg stand and catch training may enhance dynamic balance by continuously challenging postural control during functional tasks, potentially contributing to improved ankle stability.

### 4.2 Impact of PNF training on CAIT scores

Although FAI has been extensively investigated, no single evaluation method has been universally accepted as the definitive standard. The CAIT questionnaire has been widely used and validated in clinical and sports rehabilitation research to assess perceived ankle instability. In the present study, both groups demonstrated significant improvements in CAIT scores after the intervention, with greater improvements observed in the PNF training group. These findings are consistent with previous studies reporting beneficial effects of PNF-based interventions on functional ankle outcomes. For example, Yesilkir et al. found that PNF training resulted in greater improvements in postural control and neuromuscular coordination compared with standard physiotherapy in individuals with ankle instability (40). Similarly, Harmouche et al. reported improvements in functional outcomes and perceived ankle stability following PNF intervention (41). The consistency between these findings may be explained by the fact that both previous studies and the present study applied PNF principles emphasizing coordinated movement patterns, resistance-based activation, and functional task practice, which may contribute to improvements in perceived ankle function. However, differences in participant characteristics, intervention protocols, and outcome measures should be considered when interpreting these findings.

The CAIT questionnaire primarily reflects subjective perceptions of ankle stability and symptoms experienced during daily and physical activities. Improvements in CAIT scores after PNF training may therefore reflect enhanced functional performance and sensorimotor control during ankle-related tasks. Although proprioception was not directly assessed in the present study, the observed improvements may be related to enhanced proprioceptive input and neuromuscular coordination associated with PNF-based training. By incorporating spiral-diagonal movement patterns and multi-joint functional movements, PNF may provide greater challenges to integrated lower-limb control than isolated ankle training, which could partially explain the greater improvements observed in perceived ankle stability.

### 4.3 Impact of PNF training on balance ability

Balance relies on sensory input from vision, the vestibular system, and proprioceptors, and the integrative coordination of the central nervous system. This study evaluated the impact of PNF training on balance ability in habitually active adults by comparing center of gravity movement track tests and star excursion balance tests before and after training. The center of gravity movement track test accurately measures sway frequency and stability coefficient, effectively reflecting balance ability.

PNF techniques incorporate proprioceptive stimulation, resisted movement, and multi-joint coordination, which may provide enhanced sensorimotor challenges during functional tasks. These training components may contribute to improved sensorimotor integration across the lower limb, although proprioceptive changes were not directly measured in the present study. During PNF training, the therapist continuously contacts the participant's skin to help them sense muscle movement directions, enhancing skin sensation. Quick pulling of the ankle joint induces muscle stretch reflexes, mobilizing weak muscles to contract quickly, enhancing their strength and reaction speed. Providing maximum resistance encourages both agonist and antagonist muscles to work, improving muscle endurance, coordination, and balance. Therapists' verbal cues during training stimulate voluntary movements, and visual focus on limb movements enhances neuromuscular control. PNF training significantly improved balance ability in participants with functional ankle instability, as reflected in balance outcomes (54). These results are consistent with prior findings showing that proprioceptive and PNF-based interventions effectively enhance postural control and neuromuscular coordination (55). Lazarou et al. reported that individuals who underwent PNF training after ankle sprain demonstrated significant improvements in dorsiflexion range of motion and functional balance performance 8 weeks post-intervention, supporting the current study's results (56). Together, these findings indicate that PNF-based approaches facilitate proprioceptive feedback, joint control, and balance restoration across different populations.

The results of the current study support the potential role of PNF-based training in improving balance performance, possibly through enhanced sensorimotor coordination and functional movement control.

### 4.4 Impact of PNF training on ankle muscle strength

PNF is theoretically designed to enhance neuromuscular coordination through proprioceptive stimulation, resisted movement patterns, and coordinated activation of agonist and antagonist muscles. These mechanisms may contribute to improved muscle activation efficiency and force production during functional movements. The physiological basis of PNF has been associated with neurophysiological concepts such as autogenic inhibition and reciprocal inhibition, in which sensory input from muscle spindles and Golgi tendon organs may influence muscle activation and relaxation responses (53). Isometric contractions used in PNF may provide strong sensory stimulation and facilitate neuromuscular adaptation through repeated activation of targeted muscle groups. In addition, coordinated contraction of agonist and antagonist muscles during PNF patterns may contribute to improved movement control and functional muscle performance (57).

The findings of this study align with previous evidence supporting the efficacy of PNF-based strength training in improving ankle muscle function among individuals with chronic ankle instability. Hall et al. conducted a randomized controlled trial involving 39 participants with chronic ankle instability, who were assigned to a resistance-band training group, a PNF strength-protocol group, or a control group. After 6 weeks of intervention, both training groups showed significant improvements in ankle muscle strength and perceived stability compared with the control group. Notably, the PNF group demonstrated significant gains in inversion and eversion strength, indicating that PNF facilitates multidirectional muscle activation through spiral-diagonal movement patterns. Consistent with these findings, the present study demonstrated significant pre–post improvements in ankle dorsiflexion, plantarflexion, inversion, and eversion strength following PNF training. However, these improvements were not universally superior to those observed in the strength-plus-balance training group. PNF lower limb spiral–diagonal movement patterns involve coordinated activation of ankle dorsiflexors, plantarflexors, invertors, and evertors through multi-joint motions. These patterns emphasize synergistic muscle recruitment across the ankle, knee, and hip, promoting balanced activation of agonist and antagonist muscle groups rather than isolated strengthening of individual muscles. This integrated neuromuscular activation may partially contribute to the observed improvements in overall ankle muscle strength following PNF training (47).

The results indicated no significant differences between PNF training and strength-plus-balance training in enhancing ankle dorsiflexion, plantarflexion, and eversion strength. However, strength-plus-balance training was more effective in improving ankle inversion strength. This between-group difference may be attributable to the greater movement specificity and consistent mechanical loading applied to the invertor muscles during resistance-band training, which targeted inversion directly in every session. In contrast, PNF training, while effective in enhancing overall neuromuscular coordination and global ankle strength, involved relatively fewer inversion-dominant tasks within the selected movement patterns, which may have limited its effect on maximal inversion strength development.

Functional recovery in individuals with FAI is a complex process involving the interaction of perceived stability, postural control, and muscle function. In the present study, improvements in CAIT scores may reflect enhanced confidence and functional capacity during ankle-related activities, whereas changes in balance performance indicate improvements in the ability to maintain postural control under static and dynamic conditions. Meanwhile, alterations in ankle muscle strength represent the physical capacity required to support joint stability and movement execution. These functional domains are closely interconnected, as adequate muscle performance contributes to effective postural adjustments, while improved sensorimotor coordination may facilitate the integration of strength and balance during functional tasks. The greater improvements in CAIT scores and balance outcomes observed following PNF-based training, together with comparable improvements in ankle muscle strength, suggest that PNF may provide broader benefits in restoring integrated functional control. In contrast, the superior improvement in inversion strength observed after strength-plus-balance training highlights the potential value of task-specific strengthening for addressing individual muscle deficits. Therefore, different rehabilitation approaches may influence distinct components of functional recovery, and exercise selection should consider the primary functional limitations of individuals with FAI.

### 4.5 Limitations

This study has several limitations. First, participants were university students majoring in sports, who may differ from competitive athletes, sedentary individuals, or patients with chronic mechanical ankle instability; therefore, the generalizability of the findings to other populations should be interpreted with caution. Second, the intervention period was limited to 8 weeks, and no long-term follow-up assessment was conducted. Future studies with extended follow-up are needed to determine the durability of the observed effects. Third, although multiple functional domains, including perceived ankle stability, balance performance, and muscle strength, were assessed, the current analysis focused on individual outcomes and did not further examine interactions among these domains during recovery. Fourth, direct neurophysiological assessments, such as electromyography or neuroimaging, were not included; therefore, the mechanisms underlying the observed improvements could not be directly verified. Finally, although an active comparison intervention was used, the absence of a passive control group and the reliance on self-reported CAIT scores may limit interpretation of the absolute effects and introduce potential response bias. Future studies involving larger and more diverse samples, longer follow-up periods, and integrated mechanistic assessments are warranted.

## 5 Conclusion

Habitually active adults with FAI exhibited impaired balance stability and reduced muscle strength on the affected side compared with the contralateral side. Both PNF training and strength-plus-balance training improved functional outcomes after the 8-week intervention. PNF training may provide greater improvements in perceived ankle stability, postural stability, and dynamic balance performance, whereas strength-plus-balance training may be more beneficial for improving specific ankle muscle strength, particularly inversion strength. These findings suggest that PNF and strength-plus-balance training may have distinct but complementary effects in the rehabilitation of FAI. PNF may be considered as part of rehabilitation programs when the primary goals include improving perceived ankle stability and balance performance, while strength-plus-balance training may be appropriate when targeting specific strength deficits.

## Data Availability

The raw data supporting the conclusions of this article will be made available by the authors, without undue reservation.
